# Comparative evaluation of phytate and phytase addition on growth performance and nutrient utilization efficiency in broilers and ducks

**DOI:** 10.1016/j.psj.2026.106637

**Published:** 2026-02-12

**Authors:** Shoaib Ahmed Pirzado, Zheng Aijuan, Chen Jiang, Zou Zhiheng, Liu Guohua

**Affiliations:** a Institute of Feed Research, Key Laboratory for Feed Biotechnology of the Ministry of Agriculture and Rural Affairs, Chinese Academy of Agriculture Sciences, Beijing 100081, China; b Department of Animal Nutrition, Sindh Agriculture University, Tandojam, 70060, Pakistan; c Institute of Animal Husbandry and Veterinary Science, Jiangxi Academy of Agricultural Sciences, Nanchang 330200, China

**Keywords:** Phytate, Phytase, Broilers and Ducks, Growth, Nutrient utilization

## Abstract

This study aimed to comparative evaluation of dietary phytate level and phytase addition on growth performance and nutrient utilization efficiency in 21 day-aged broilers and ducks. A total of 540 one-day-old male Arbor-Acres broilers and 540 Cherry Valley ducks were randomly assigned to 9 dietary treatments, with 6 replicates of 10 birds each (60 birds per treatment in total). The dietary treatments included a positive control (PC) containing 0.40% non-phytate phosphorus (nPP) without phytase, and 8 low nPP diets (0.24% nPP) arranged in a 2 × 4 factorial design. The factorial treatment consisted of two phytate-P levels (2.4 and 3.4 g/kg) and four phytase supplementation levels (0, 500, 1000, and 1500 FTU/kg).

The results showed that compared with the PC, feeding low-nPP diets significantly impaired the growth performance of both broilers and ducks (*P* < 0.05). In the absence of phytase, high phytate-P exerted a negative effect on the growth performance in broilers but significantly improved that of ducks (*P* < 0.05). For broilers, low phytate-P significantly enhenced calcium (Ca) and phosphorus (P) retention, as well as apparent metabolizable energy (AME). In contrast, high phytate-P reduced Ca retention without affecting P retention or AME. For ducks, both phytate-P levels of reduced Ca and P retention, but high phytate-P augured a significant increase in AME (*P* < 0.05). Under low-nPP diets, plasma P levels decreased in both species; in broilers, plasma P was further reduced by high phytate-P, whereas ducks showed the opposite pattern (*P* < 0.05). Plasma Ca remained unchanged in broilers but increased with elevated phytate-P in ducks (*P* < 0.05). Phytase supplementation significantly improved growth performance, nutrient retention, and plasma P levels in both species (*P* < 0.05). In addition, phytase inclusion increased AME in ducks alon, with no significant effects on AME in broilers or on plasma Ca levels in either species. Collectively, these findings demonstrate species-specific responses of broilers and ducks to dietary phytate and phytase, indicating that phosphorus utilization data derived from broilers cannot be directly extrapolated to ducks. Therefore, species-specific adjustments should be made in feed formulations to optimize nutrient utilization and growth performance in each species.

## Introduction

Phosphorus (P) has a substantial role in poultry nutrition, particularly in skeletal development, energy metabolism, and other physiological functions. However, around two-thirds of the total phosphorus found in plant-derived feedstuffs is in the form of phytate-bound phosphorus (phytate-P) (Punna and Roland Sr, 1999; Viveros et al., 2000). Monogastric animals have low levels of phytases, so they cannot effectively digest phytate, which makes phytate-P difficult to absorb. Phytate-P is very much intractable for absorption and hence leads to poor utilization of phosphorus. Besides phytate adversely affecting the bioavailability of phosphorus, it has other antinutritional features. Phytate forms insoluble complexes with protein, lipids, and starch, which diminishes the digestibility of these macronutrients and increases the extent of non-response growth (Ravindran et al., 1995; Cowieson et al., 2006). Moreover, it also reduces absorption of divalent cations such as calcium (Ca²⁺), manganese (Mn²⁺), zinc (Zn²⁺), copper (Cu²⁺), and magnesium (Mg²⁺) thus causing a reduction in essential nutrients (Cosgrove and Irving, 1980; Thompson and Yoon, 1984). All these factors have been shown to increase production losses and reduce efficiency (Liu et al., 2007, 2008). In addition, there is evidence that dietary phytate increases amino acid losses, increasing the nutrient inefficiency of the diet (Cowieson et al., 2004; Cowieson and Ravindran, 2007).

Phosphorus utilization from plant-based diets in ducks ranged from 28% to 49%, while in broilers it was between 40% to 73% (Rodehutscord and Dieckmann, 2005). It must be noted that these values show the percentage of total phosphorus present in the feedstuffs listed have an exceptionally high concentration of phytate phosphorus: maize (97.2%), sorghum (97.2%), rice bran (87.8%), soybean meal (84.4%), and wheat (100%) of total inositol phosphate (Kasim and Edwards, 1998). Phosphorus bioavailability in corn-soy diets, which are the mainstay in commercial poultry production, is often low. Thus, inorganic phosphorus supplements are necessary. While meeting nutritional requirements, this approach raises feed costs and contributes to excess phosphorus pollution through faecal excretion of unutilized phytate-P.

Phytase is an enzyme that hydrolyses phytate and liberates inorganic phosphate groups, facilitating the release of phosphorus and other nutrients in monogastric animals. There are three main sources from which phytase can be obtained: intrinsic phytase found in some feed constituents (Eeckhout and De Paepe, 1994; Viveroset al., 2000), endogenous phytase from the intestinal flora and the intestinal microvilli (Pallauf et al., 1994) and exogenous phytase added to the feed. Out of these, the endogenous and ingredient-based phytase seems to be less than sufficient to cover the nutritional demands of the animals. Hence, the supplementation of microbial phytase has become the standard method to enhance phosphorus and mineral utilization, as well as the performance of poultry, without fearing adverse consequences for their welfare (Dilger et al., 2004; Haile et al., 2020; Ravindran et al., 2001).

Some studies have shown that when fed soybean meal, ducks may utilize energy more efficiently than broilers (Mohamed et al., 1984). The two species also differ concerning the digestibility of amino acids: in a comparative study, the mean amino acid digestibility at 14, 28, and 42 days of age yielded 69.7%, 73.0%, and 72.5% in broilers and 43.8%, 61.8%, and 59.8% in ducks, respectively, provided identical diets (Jamroz et al., 2002). Regardless of these findings, very few studies have been conducted to directly compare nutrient utilization, especially for calcium, phosphorus, and energy, between broilers and ducks kept under the same diet and housing conditions. The focus of this research is on determining how both species respond to high-phytate diets with or without exogenous phytase supplementation.

This study was undertaken to assess and compare the impacts of high-phytate diets and exogenous phytase on calcium, phosphorus, and energy utilization in broiler chickens and ducks. To enhance the understanding of interspecies variations in response to phytate and phytase, both species were maintained under the same housing and management conditions and provided the same diets.

## Materials and methods

### Ethics statement

All animal experiments were performed in compliance with the guidelines of the National Commission for the care and use of laboratory animals. The experimental procedures were conducted in accordance with the protocol reviewed and approved by the Ethics Committee of the Chinese Academy of Agricultural Sciences (Statement No. AEC—CAAS20250122).

### Phytase

The phytase used in this trial was a commercially available product (XP-TPT 10000 G; Danisco Animal Nutrition), derived from *Escherichia coli*. The analyzed enzyme activity was 10,134 U/g. One unit of phytase activity (U) was defined as the amount of enzyme that releases 1 µmol of inorganic phosphate per minute from 5.1 mmol/L sodium phytate at pH 5.5 and 37°C, under standard assay conditions in a water bath.

### Experimental design and dietary treatments

The study was conducted based on a 2 × 4 factorial design which consisted of two levels of dietary phytate-P: 2.4 g/kg and 3.4 g/kg, alongside four levels of supplemental microbial phytase at 0, 500, 1,000, and 1,500 FTU/kg. Moreover, a positive control group was added which was formulated with 4.0 g/kg of non-phytate phosphorus (nPP) as a reference. All diets were prepared based on corn, soybean meal, cotton seed meal, rapeseed meal and wheat bran. All these ingredients were analyzed for their contents of calcium, total phosphorus, and phytic acid phosphorus prior to use. Detailed dietary compositions along with the nutrient levels are provided in Table 1.

**Table 1:** Nutrient composition of basal diet.

Ingredient %	Control	Low Phytate-P	High Phytate-P
Corn	49.34	50.02	42.30
Soybean oil	4.63	4.41	5.04
Soybean meal	30.76	30.63	29.73
Cotton seed meal	4	4	4
Rice bran	3	3	11
Canola	4	4	4
Limestone	1.42	2.03	2.01
CaHPO_4_	1.27	0.33	0.36
Lys	0.12	0.12	0.1
Met	0.16	0.16	0.16
NaCl	0.3	0.3	0.3
Premix (1%)^1^	1	1	1
Nutrient Levels			
AME, Mcal/Kg^2^	2.95	2.95	2.95
Crude protein, %^3^	20.87	20.91	21.12
Starch, % 3	49.80	52.32	47.16
Crude fat, %^3^	7.67	7.50	8.34
Calcium, %^3^	0.94	0.95	0.94
Total phosphorus, %^3^	0.65	0.49	0.60
Non-phytate phosphorus,%^2^	0.40	0.25	0.25
Phytate phosphorus, %^3^	0.25	0.24	0.35
Lysine, %^3^	1.11	1.12	1.11
Methionine, % 3	0.49	0.49	0.49
Met+Cys, %^3^	0.85	0.85	0.85
Threonine, %^3^	0.85	0.85	0.85
Tryptophan, %^3^	0.28	0.28	0.28
D. Lys, %^2^	0.92	0.92	0.89
D. Methionine, %^2^	0.42	0.42	0.41
D. Methionine+Cystine,%^2^	0.71	0.71	0.70
D. Threonine^2^	0.71	0.71	0.69
D. Tryptophan^2^	0.24	0.24	0.23

1 Premix supplied the following per kilogram of diet: Vitamin A, 2500 IU;Vitamin D, 3400IU;Vitamin E, 10 IU;Vitamin K, 30.5 mg;Vitamin B1, 1.8 mg;Vitamin B2, 4.0 mg;Vitamin B6, 3.0 mg; Vitamin B12, 710 μg; pantothenic acid, 11 mg; niacin, 55 mg, folic acid, 0.5 mg; biotin, 0.12 mg, choline chloride, 750 mg,; Cu, 8 mg; Fe, 80 mg, Zn, 40 mg; Mn, 60 mg; Se, 0.15 mg; I, 0.35 mg.

2 Calculated values.

3 Analytical values.

### Animal and feeding management

A total of 540 one-day-old AA+ male broiler chicks and 540 one-day-old Cherry Valley male ducklings, all in good health and with comparable body weights, were selected for the experiment. The birds were randomly allocated into 9 treatment groups, with 6 replicates per group and 10 individuals per replicate. The trial duration was 21 days. The experimental animals were housed in stacked cages under a 18-hour lighting regime, with ad libitum access to drinking water and feed. Avian influenza vaccination was administered at 10 days of age. For the experimental diet, mash feed was provided in the first week, while pelleted feed (processed through pelleting machine) was used in the subsequent two weeks.

### Collection of samples and measurements

Live body weight was recorded on a cage basis at the beginning (day 1) and end of the trial (day 21). Daily monitoring including feed intake, morbidity, and mortality was documented. Subsequently, live body weight (LBW), average daily gain (ADG), average daily feed intake (ADFI), and feed-to-gain (F/G) ratio were calculated.

Titanium dioxide (TiO₂) was added to the experimental diets at a concentration of 4 g/kg as an indigestible marker. For each metabolic cage, clean plastic sheets were placed under the traps to collect excreta. Excreta were collected every day at 08:00 from day 19 to 21. The samples of excreta were mixed thoroughly, then subsampled in specific ratios which were preserved at –20 °C until further analysis. At the end of the collection period, samples were thawed and pooled by replicate before being heated to 105°C for 15 minutes and then dried to constant weight at 65°C in forced air ovens. Both diet and excreta samples were ground to pass through a 0.45 mm sieve before chemical analysis.

The dietary nPP of is obtained by subtracting the value of phytate-P from that of total phosphorus. The content of phytate-P was determined in accordance with ISO 3123:2020. Titanium dioxide (TiO₂), dry matter (DM), nitrogen (N), gross energy (GE), calcium (Ca), and phosphorus (P) contents in both diets and excreta were analyzed to determine the apparent utilization efficiency of DM, nitrogen, energy, calcium, and phosphorus. DM and nitrogen content were determined using the oven-drying method and the Dumas method, respectively. TiO₂ concentration was measured according to the method described by (Xu et al., 2021), while gross energy was determined using an oxygen bomb calorimeter (IKA Calorimeter C200). Dried feed and excreta samples were first carbonized on an electric heating panel until reduced to grey ash, then incinerated in a muffle furnace at 550 °C for 5 hours. After cooling, the ash was dissolved in 10 mL of 6 N HCl by heating, followed by filtration. The resulting filtrate was used to determine calcium and phosphorus concentrations. Calcium content was quantified using a flame atomic absorption spectrophotometer (AAS 400P), and phosphorus content was measured using the vanadium molybdate yellow colorimetric method. The apparent utilization of energy, calcium and phosphorus was calculated according to the following equation:

$$Apparent\;nutrient\;utilization\;\left(\%\right)\;=100\;\times \;\left[1-\left(\frac{TiO_{2\;diet}}{TiO_{2\;excreta}}\times \frac{Nutrient_{excreta}}{Nutrient_{diet}}\right)\right]$$

where TiOs and Nutrient are based on DM basis.

From every replicate, two birds were chosen and slaughtered for blood sampling based on the average body weight. About 2 mL blood sample was obtained and placed in heparinized tubes. Subsequently, plasma was separated by centrifugation at 3000 rpm for 15 minutes and stored at −20°C until analysis. Plasma calcium (Ca) and phosphorus (P) concentrations were measured using commercial colorimetric assay kits from Randox Laboratories Ltd. (Crumlin, County Antrim, United Kingdom). The absorbance was then recorded with a BA-88 Semi-Automatic Chemistry Analyzer (BioSystems S.A., Barcelona, Spain), following the manufacturer's instructions.

## Statistical analysis

The statistical analysis was performed using SPSS (version 16.0; SPSS Inc., Chicago, IL, USA). The two factors of phytate-P and phytase supplementation, along with their interaction, were analyzed by two-way ANOVA under the General Linear Model (GLM) procedure. Control treatment was not included in the factorial analysis. Significant differences (*P* < 0.05) were measured by Duncan’s multiple range test. Results are presented as means ± standard deviation (SD).

## Results

### Growth performance

The effects of phytate-P levels and phytase supplementation on growth performance of the two species are depicted in Table 2. The addition of phytase significantly (*p* < 0.001) improved the LBW, ADG, ADFI and F/G in both broilers and ducks. Ducks showed higher LBW, ADG and ADFI compared to broilers in all treatments. The interaction between phytate-P and phytase inclusion had significantly improved LBW, ADG, ADFI, and F/G in both broiler and ducks, revealing that phytase was more efficient in boosting weight gain at lower at phytate-P levels. The maximum ADG *(p* < 0.001) was noticed in broilers at 2.4 g/kg of phytate-P with 1000 FTU/kg phytase supplementation. The ducks obtained higher ADG *(p* < 0.001) at 3.4 g/kg phytate-P at 1500 FTU/kg. However, LBW and ADG was lower compared to control treatment without phytase. Moreover, the better F/G was observed at 2.41 g /kg phytate-P at 1000 FTU /kg phytase in both broilers and ducks. The interaction between phytate-P and phytase shown that broilers and ducks got more benefit from phytase at low phytate-P (2.14 g/kg) levels.

**Table 2:** Effects of dietary phytate-P levels with and without phytase supplementation on growth performance of broilers and ducks.

Phytate-P (g/kg)	Phytase (FIU/kg)	Broilers				Ducks			
		LBW (g)	ADG (g/d)	ADFI (g/d)	F/G	LBW (g)	ADG (g/d)	ADFI (g/d)	F/G
PositiveControl	0	677	30.18^a^	58.73^a^	2.00^de^	1140	51.71^a^	112.27^a^	2.19^e^
2.4^1^	0	508	22.14^c^	52.89^cd^	2.66^a^	694	30.49^e^	93.94^f^	3.23^a^
	500	693	30.95^a^	58.85^a^	1.92^e^	900	40.28^cd^	100.92^e^	2.54^cd^
	1000	731	32.76^a^	60.36^a^	1.88^e^	1051	47.47^b^	108.09^abc^	2.29^e^
	1500	700	31.28^a^	59.55^a^	1.94^e^	1019	45.94^b^	107.39^bcd^	2.38^de^
	SEM	2	0.10	0.22	0.02	5	0.26	0.56	0.01
	ANOVA *P*	0.040	0.041	0.023	0.019	0.004	0.005	0.007	<0.001
	Linear *P*	0.064	0.065	0.011	0.082	0.078	0.079	0.002	0.087
	Quadratic *P*	0.021	0.021	0.043	0.015	0.038	0.037	0.565	0.034
3.4^1^	0	516	22.54^bc^	52.74^d^	2.55^a^	846	37.72^d^	103.78^de^	2.86^b^
	500	542	23.77^bc^	55.19^bc^	2.46^ab^	940	42.18^c^	105.81^cd^	2.57^cd^
	1000	567	24.95^bc^	55.88^b^	2.28^bc^	937	42.04^c^	108.80^abc^	2.71^bc^
	1500	589	26.02^b^	56.22^b^	2.21^cd^	1050	47.42^b^	110.57^ab^	2.40^de^
	SEM	3	0.15	0.23	0.03	8	0.36	0.59	0.02
	ANOVA *P*	0.004	0.004	0.005	0.007	0.005	0.006	0.005	0.005
	Linear *P*	0.022	0.025	0.034	0.019	0.011	0.014	0.028	0.061
	Quadratic *P*	0.118	0.128	0.048	0.111	0.237	0.239	0.302	0.047
Main effects^2^									
Phytate-P	2.4g/kg	658	29.28^A^	57.91^A^	2.10^B^	916	41.05^B^	102.58^B^	2.61
	3.4g/kg	554	24.32^B^	55.00^B^	2.37^A^	943	42.34^A^	107.24^A^	2.63
	SEM	2	0.08	0.17	0.01	3	0.12	0.71	0.01
	ANOVA *P*	<0.001	<0.001	<0.001	<0.001	0.061	0.064	<0.001	0.622
Phytase	0	512	22.34^B^	52.82^B^	2.60^A^	770	34.11^C^	98.86^B^	3.05^A^
	500	618	27.36^A^	57.02^A^	2.19^B^	920	41.23^B^	103.36^B^	2.55^B^
	1000	649	28.86^A^	58.12^A^	2.08^B^	994	44.76^A^	108.44^A^	2.50^BC^
	1504	645	28.65^A^	57.88^A^	2.08^B^	1034	46.68^A^	108.98^A^	2.39^C^
	SEM	2	0.09	0.20	0.01	4	0.17	0.66	0.02
	ANOVA *P*	<0.001	<0.001	<0.001	<0.001	<0.001	<0.001	<0.001	<0.001
	Linear *P*	0.140	0.138	0.244	0.303	0.203	0.198	0.121	0.050
	Quadratic *P*	0.045	0.045	0.034	0.054	0.021	0.019	0.023	0.020
Phytate*Phytase	*P*	0.012	0.011	0.062	0.003	<0.001	<0.001	0.020	<0.001

Means with the same row having different superscripts (a, b, c for ANOVA; A, B, C for MANOVA) differ significantly (*P* < 0.05). LBW, live body weight; ADG, average daily gain; ADFI, average daily feed intake; F/G, Feed to gain.

### Utilization of DM, nitrogen, calcium, phosphorus and energy

The effects of phytate-P levels and phytase supplementation on nutrition utilization of the two species are presented in Table 3. Dietary treatment had no significant effect on DM and nitrogen utilization in the two species (*p* > 0.05). However, the P and Ca retention were significantly (*p* < 0.001) improved by Phytate-P level and phytase inclusion. Ducks exhibited maximum P and Ca digestibility among all treatment groups in comparison with broilers. The interaction between phytate-P and phytase supplementation had a significant (*p* < 0.01) effect for P and Ca digestibility, revealing that the combined effect of low level of phytate-P and high phytase dose resulted maximum nutrient utilization. The highest P digestibility (62.95) at 2.4 g/kg phytate with 1500 FTU /kg was observed in broilers and similar trend was noticed in ducks. Similarly, in ducks Ca digestibility recorded maximum (49.31) at 3.4 g /kg with 1500 FTU /kg phytase. Whereas broilers exhibited highest Ca digestibility (67.13) at 2.4 g/kg phytate-P with similar phytase dose. AME digestibility improved in ducks compared to broilers. The ducks obtained highest AME digestibility (14.70 MJ/Kg) at 3.4 g /kg phytate-P combined with 1500 FTU g/kg phytase, while broilers attained (13.71 MJ/kg) at 2.4 g/kg phytate-P without addition of phytase. The interaction between phytate-P and phytase showed significant (*p* < 0.01) effect on AME digestibility.

**Table 3:** Effects of dietary phytate-P levels with and without phytase supplement on nutrients utilization of broilers and ducks.

Phytate-P (g/kg)	Phytase (FIU/kg)		Broilers					Ducks			
		DM %	N (%)	P (%)	Ca (%)	AME (MJ/kg)	DM (%)	N (%)	P (%)	Ca (%)	AME (MJ/kg)
Control	0	70.02	77.87	44.74^e^	53.52^c^	12.97^c^	73.06	80.32	42.53^b^	52.83^a^	14.26^def^
2.4^1^	0	73.34	77.21	52.52^bcd^	62.90^ab^	13.71^a^	75.27	78.40	34.54^f^	31.88^f^	14.36^cde^
	500	71.33	76.27	62.34^a^	67.69^a^	13.36^b^	74.03	78.95	41.26^bc^	29.81^f^	14.08^f^
	1000	69.56	75.37	56.23^b^	65.37^ab^	13.22^bc^	75.19	81.36	38.19^de^	36.97^e^	14.24^ef^
	1500	69.14	76.22	62.95^a^	67.13^a^	13.18^bc^	76.04	78.59	47.32^a^	42.16^d^	14.44^cd^
	SEM	0.69	0.50	0.33	0.29	0.04	0.23	0.94	0.27	0.29	0.05
	ANOVA *P*	0.112	0.617	0.002	<0.001	0.011	0.170	0.122	0.003	<0.001	0.064
	Linear *P*	0.046	0.331	0.544	0.631	0.045	0.043	0.137	0.656	0.087	0.311
	Quadratic *P*	0.504	0.376	0.328	0.090	0.064	0.007	0.204	0.210	0.078	0.052
3.4^1^	0	69.82	76.83	44.09^e^	45.28^d^	12.94^c^	74.48	80.06	35.82^ef^	41.66^d^	14.49^bc^
	500	68.25	75.29	46.93^de^	59.78^abc^	13.33^b^	74.66	80.72	36.86^def^	46.67^bc^	14.68^ab^
	1000	70.11	75.71	50.84^cd^	58.15^bc^	13.43^ab^	74.65	80.96	39.34^cd^	43.73^cd^	14.64^ab^
	1500	68.82	76.94	52.56^bc^	60.86^abc^	13.48^ab^	75.50	82.36	43.06^b^	49.31^b^	14.70^a^
	SEM	0.58	0.57	0.13	0.20	0.03	0.33	0.40	0.13	0.17	0.02
	ANOVA *P*	0.675	0.705	<0.001	<0.001	<0.001	0.756	0.265	0.002	<0.001	0.002
	Linear *P*	0.806	0.889	0.023	0.046	0.031	0.348	0.054	0.033	0.025	0.041
	Quadratic *P*	0.835	0.372	0.768	0.890	0.689	0.601	0.612	0.676	0.673	0.703
Main effects^2^											
Phytate-P	2.4g/kg	70.90	76.72	58.51^A^	65.78^A^	13.37	75.18	79.38	40.33^A^	35.21^B^	14.28^B^
	3.4g/kg	69.21	76.19	48.61^B^	56.11^B^	13.30	74.79	80.96	38.77^B^	45.34^A^	14.63^A^
	SEM	0.62	0.69	0.31	0.27	0.02	0.28	0.42	0.26	0.25	0.03
	ANOVA *P*	0.078	0.590	<0.001	<0.001	0.521	0.434	0.009	<0.001	<0.001	0.042
Phytase	0	71.58	77.96	48.31^C^	54.09^B^	13.33	74.88	79.30	35.18^C^	36.77^C^	14.42^B^
	500	69.79	76.27	54.63^B^	63.73^A^	13.35	74.37	79.92	39.06^B^	38.24^C^	14.38^B^
	1000	69.83	75.37	53.53^B^	61.76^A^	13.33	74.92	81.16	38.76^B^	40.35^B^	14.44^AB^
	1500	68.98	76.22	57.76^A^	63.99^A^	13.33	75.80	80.30	45.19^A^	45.74^A^	14.57^A^
	SEM	0.86	0.97	0.32	0.41	0.03	0.39	0.61	0.29	0.30	0.03
	ANOVA *P*	0.199	0.309	<0.001	<0.001	0.612	0.118	0.153	<0.001	<0.001	0.046
	Linear *P*	0.055	0.162	<0.001	<0.001	0.418	0.079	0.419	0.025	<0.001	<0.001
	Quadratic *P*	0.579	0.169	<0.001	0.004	0.997	0.067	0.250	<0.001	<0.001	0.046
Phytate*Phytase	*P*	0.292	0.445	0.016	0.193	<0.001	0.607	0.135	0.002	<0.001	0.009

Means with the same row having different superscripts (a, b, c for ANOVA; A, B, C for MANOVA) differ significantly (*P* < 0.05). DM, dry matter; N, nitrogen; P, phosphorus; Ca, calcium; AME, apparent metabolizable energy.

### Plasma phosphorus and calcium

The effects of dietary phytate-P levels and phytase supplementation on plasma phosphorus (P) and calcium (Ca) concentrations in broilers and ducks are summarized in Table 4. Phytase supplementation resulted in a significant increase (*p* < 0.05) in plasma P levels in both species. Overall, ducks exhibited higher plasma P concentrations compared to broilers, particularly at elevated phytase inclusion rates. A significant interaction (*p* < 0.05) between phytate-P and phytase was detected for plasma P. Specifically, in broilers, plasma P increased by 2.03% with 1500 FTU/kg phytase at 2.4 g/kg phytate-P, while in ducks, plasma P increased by 2.53% under the same phytase level at 3.4 g/kg phytate-P. Conversely, plasma Ca concentrations in broilers did not differ significantly across treatments. The highest plasma Ca concentration (3.05%) was observed in the un supplemented group at 2.14 g/kg phytate-P. No significant interaction (*p* > 0.05) between phytate-P and phytase was found for plasma Ca concentrations.

**Table 4:** Effects of dietary phytate-P levels with and without phytase supplement on plasma P and plasma Ca of broilers and ducks.

Phytate-P (g/kg)	Phytase (FIU/kg)	Broilers		Ducks	
		plasma P (mmol/L)	plasma Ca (mmol/L)	plasma P (mmol/L)	plasma Ca (mmol/L)
Control	0	2.12^a^	2.38	2.58^a^	2.93^ab^
2.4^1^	0	1.40^f^	2.40	1.25^c^	3.05^a^
	500	1.78^bcd^	2.37	1.38^c^	2.87^abc^
	1000	1.91^abc^	2.23	2.11^b^	2.87^abc^
	1500	2.03^ab^	2.20	2.24^ab^	2.85^bc^
	SEM	0.02	0.01	0.02	0.03
	ANOVA *P*	0.010	0.208	0.009	0.023
	Linear *P*	0.046	0.117	0.152	0.349
	Quadratic *P*	0.048	0.050	0.025	0.011
3.4^1^	0	1.42^ef^	2.43	1.47^c^	2.73
	500	1.50^de^	2.48	1.99^b^	2.70
	1000	1.67^cde^	2.20	2.04^b^	2.83
	1500	1.98^ab^	2.22	2.53^a^	2.82
	SEM	0.01	0.02	0.02	0.03
	ANOVA *P*	0.002	0.787	0.003	0.326
	Linear *P*	0.015	0.452	0.115	0.344
	Quadratic *P*	0.106	0.291	0.031	0.469
Main effects^2^					
Phytate-P	2.4g/kg	1.78^A^	2.30	1.74^B^	2.91^A^
	3.4g/kg	1.64^B^	2.33	2.00^A^	2.77^B^
	SEM	0.02	0.01	0.02	0.03
	ANOVA *P*	0.043	0.649	0.012	0.006
Phytase	0	1.41^C^	2.42	1.36^D^	2.89
	500	1.64^B^	2.43	1.68^C^	2.78
	1000	1.79^B^	2.22	2.08^B^	2.85
	1500	2.00^A^	2.21	2.39^A^	2.83
	SEM	0.02	0.02	0.02	0.03
	ANOVA *P*	0.005	0.056	<0.001	0.458
	Linear *P*	0.002	0.591	0.004	0.600
	Quadratic *P*	0.219	0.084	0.231	0.718
Phytate*Phytase	*P*	0.336	0.907	0.132	0.129

Means with the same row having different superscripts (a, b, c for ANOVA; A, B, C for MANOVA) differ significantly (*P* < 0.05). P, phosphorus; Ca, calcium.

## Discussion

It is generally recognized that broilers have higher nutritional requirements than meat ducks. For instance, the apparent metabolizable energy (AME) contents in the starter diets of meat ducks and broilers are typically 2.90 and 2.95 Mcal/kg, respectively; the corresponding crude protein (CP) levels are 20% and 21.5%, and the non-phytate phosphorus (NPP) levels are 0.40% and 0.45%. In the present study, to ensure normal growth of both broilers and meat ducks, the dietary energy and protein levels were formulated to basically meet the requirements of broilers, while being slightly higher than those recommended for meat ducks. Specifically, the control diet was formulated to contain 2.95 Mcal/kg of apparent metabolizable energy and 21% of crude protein, as well as amino acids levels meeting the recommended requirements for broilers. Meanwhile, the non-phytate phosphorus level in the control diet was moderately reduced to 0.40%, which corresponds to the minimum NPP requirement for meat ducks. For the experimental diets, the levels of energy, crude protein and total amino acids were set as consistent with those in the control group, while only about 60% of the non-phytate phosphorus provided in the control group was supplied to establish a phosphorus-deficient model, to better elucidate the effects of different dietary phytate phosphorus levels and phytase supplementation. The experimental results demonstrated that the dietary nutritional levels in this study were rational, and it was sufficient to sustain normal growth of both broilers and meat ducks during the trial period.

### Growth performance

As demonstrated by the results obtained in this study, both ADG and ADFI were lower while F/G was higher in both broilers and ducks at phytate-P levels (2.4 g/kg vs 3.4 g/kg). However, the addition of phytase to the diets of broilers (1000 FTU/kg) and ducks (1500 FTU/kg) significantly improved both ADFI and ADG. Most importantly, the addition of phytase at 1000 FTU/ kg at a phytate-P level of 2.4 g/kg in both broilers and ducks led to considerable improvement in F/G. ADFI and ADG were lower and FCR was poor at both low (2.2 g/kg) and high (4.4 g/kg) phytate-P diets, but (Liuet al., 2008) showed that the addition of phytase (1000 FTU/kg) to low phytate-P (2.2 g/kg) diets yielded considerable improvements in ADFI, ADG, and F/G in broilers. Additionally, (Jamroz et al., 2001) reported that feeding ducks low phytate-P (2.00 g/kg) or high phytate-P (3.5 g/kg) supplemented with phytase increased both ADFI and ADG and improved the FCR. These results suggest that phytase supplementation to diets with phytate-P enhances growth performance in both species. These findings align with earlier research conducted on broiler chickens (Cowieson et al., 2006; Liuet al., 2007; Pirgozliev et al., 2007) and ducks (Liu Hong et al., 2010; Orban et al., 1999). The potential benefits of phytase on growth may be due to its ability to enhance the protein and starch bioavailability by liberating some minerals and other compounds which are bound with phytic acid (Camden et al., 2001; Selle et al., 2000) Additionally, it has been reported that phytase reduces the endogenous losses of some amino acids and minerals (Cowiesonet al., 2004) and also reduces the excretion of gastrointestinal mucins which improves intestinal health (Cowiesonet al., 2004; Karadas et al., 2005). It was noted that there is a different response by Broilers and Ducks to phytase doses. In broilers, the 500 FTU /kg phytase addition to low phytate-P diets restored growth performance to normal levels, while this was not achieved even at 1500 FTU/kg phytase on high phytate-P diets. Also, duck’s growth performance reached normal levels with 1000 FTU/kg phytate in low phytate-P and 1500 FTU/kg in high phytate-P diets.

These findings show that, as previously reported by other authors, phytase potentiates growth further in the context of low phytate P compared to high phytate P diets (Liu Hong et al., 2010; Liuet al., 2008; Pirzado et al., 2016). It is possible that the differences in response to dietary phytate involve differences in gut physiology, enzyme activity, nutrient needs, and attempts to ameliorate the anti-nutritional impacts of phytate must consider different species. The need to adjust the level of phytase added for each species is crucial as ducks have a higher intestinal pH, longer digesta retention time, and therefore, require more phytase to counteract the stronger inhibitory effects of phytate compared to broilers.

### Phosphorus, calcium and energy utilization

Phytase supplementation improved retention of phosphorus (P) and calcium (Ca) in both species, with greater effects in ducks. In agreement with (Olaolu, 2018), the increased dose of phytase improved mineral P digestibility, and the highest P digestibility of 62.95% was observed in broilers at 2.4 g/kg phytate-P with 1500 FTU/kg phytase. This supports earlier reports that increased phytase inclusion improves the extent of hydrolysis of phytate, thereby releasing more P for absorption (Zhang et al., 2011). Interestingly, ducks had higher Ca digestibility at 3.4 g/kg phytate-P with the same level of phytase (49.31%), which indicates that responses to enzyme supplementation may differ by species. This corroborates earlier reports suggesting that nutrient retention shows variation among poultry species due to different aspects of their digestive physiology (Pirgozlievet al., 2007). There were significant interactions between phytate-P and phytase for both P and Ca digestibility (*p* < 0.01), demonstrating the combined effect of decreasing phytate-P and increasing phytase on both P and Ca digestibility. These findings are in line with (Ahmad et al., 2000) who reported similar interactions enhancing nutrient bioavailability. In broilers, peak Ca digestibility (67.13%) was also observed with 2.4 g/kg phytate-P and 1500 FTU Phytase per kg which further demonstrates the strong effect of high phytase inclusion for digestible Ca release.

As broilers demonstrated lower energy utilization capabilities relative to waterfowl (Brenes et al., 2003), ducks surpassed them significantly in AME digestibility. Ducks showed peak AME digestibility of 14.70 MJ/kg at 3.4 g/kg phytate-P with 1500 FTU/kg phytase, and broilers reached 13.71 MJ/kg at 2.4 g/kg phytate-P with no phytase. This supports findings from (Wang et al., 2023) that reported higher energy digestibility with increased phytase supplementation. For both species, the interaction between phytate-P and phytase also significantly influenced (*p* < 0.01) AME digestibility. It indicates that increasing phytase dosage may counteract the anti-nutritive effects of phytate, thereby increasing energy utilization from the feedstuffs (Atia et al., 2000; Pirzado et al., 2024). Ducks responded most remarkably, which may stem from interspecies variation in gastrointestinal transit time and enzyme activities (Tamim et al., 2004).

### Plasma phosphorus and calcium concentrations

Phytase supplementation enhanced the plasma P concentrations of both broilers and ducks, with ducks showing a greater response. This corroborates prior studies that suggest phytase enhances the bioavailability of P, resulting in increased plasma P concentration (Fan et al., 2006). The interaction of phytate-P and phytase showed significant influence (*p* < 0.05) on plasma P concentration, with broilers reaching peak plasma P (2.03%) at 2.4 g/kg phytate-P with 1500 FTU/kg phytase, while ducks achieved 2.53% with similar phytase dosing at 3.4 g/kg phytate-P. These results are consistent with those of (Cowiesonet al., 2006), who noted that plasma P levels were enhanced by higher phytase doses. As noted, plasma Ca concentrations showed no significant alterations with the addition of phytase in broilers, where the highest levels of 3.05% were observed in the un supplemented group at 2.4 g/kg phytate-P. This is contradictory to earlier studies suggesting that phytase enhances the bioavailability of Ca (Kasim and Edwards, 1998), which points out to the possibility of other factors besides the breakdown of phytate influencing plasma Ca concentration like the calcium intake and absorption efficiency.

### Species-specific responses to phytase supplementation

The most important finding in this study is the different responses that broilers and ducks have to phytase supplementation. Ducks have always shown greater retention of nutrients and energy; this is consistent with research on interspecies differences in digestion (Pirgozlievet al., 2007). The greater improvement noted in ducks may be attributed to more efficient digestive enzyme activity and a longer intestinal retention time, which allows for more thorough hydrolysis of phytate (Cowiesonet al., 2006).

## Conclusion

In conclusion, dietary supplementation with microbial phytase significantly enhanced growth performance, nutrient digestibility, and plasma phosphorus concentration in both broilers and ducks. Broilers responded more favorably to phytase at lower phytate-P levels, whereas ducks exhibited greater improvements at higher phytate-P levels and phytase inclusion rates. Phytase addition at 1000–1500 FTU/kg proved effective in mitigating the anti-nutritional effects of phytate and enhancing phosphorus and calcium utilisation. The superior nutrient retention and energy digestibility observed in ducks suggest inherent interspecies differences in digestive physiology.

## CRediT authorship contribution statement

**Shoaib Ahmed Pirzado:** Writing – original draft, Investigation, Conceptualization. **Zheng Aijuan:** Validation, Software. **Chen Jiang:** Methodology. **Zou Zhiheng:** Formal analysis. **Liu Guohua:** Writing – review & editing, Supervision, Funding acquisition, Conceptualization.

## Disclosures

The authors have no commercial or financial relationships that could be construed as a potential conflict of interest for this project.
